# Perceptions of the healthcare providers regarding acceptability and conduct of minimal invasive tissue sampling (MITS) to identify the cause of death in under-five deaths and stillbirths in North India: a qualitative study

**DOI:** 10.1186/s12913-020-05693-6

**Published:** 2020-09-04

**Authors:** Manoja Kumar Das, Narendra Kumar Arora, Reeta Rasaily, Gurkirat Kaur, Prikanksha Malik, Mahisha Kumari, Shipra Joshi, Harish Chellani, Harsha Gaekwad, Pradeep Debata, K. R. Meena

**Affiliations:** 1 The INCLEN Trust International, New Delhi, 110020 India; 2 Division of Reproductive Biology Maternal and Child Health, Indian Council of Medical Research, New Delhi, 110029 India; 3 Department of Pediatrics, Safdarjung Hospital and Vardhman Mahavir Medical College, New Delhi, 110029 India; 4 Department of Obstetrics and Gynaecology, Safdarjung Hospital and Vardhman Mahavir Medical College, New Delhi, 110029 India

**Keywords:** Minimal invasive tissue sampling, Cause of death, Child, Neonates, Stillbirth, Healthcare providers, Hospital, Acceptance, India

## Abstract

**Background:**

India contributes the highest share of under-five and neonatal deaths and stillbirths globally. Diagnostic autopsy, although useful for cause of death identification, have limited acceptance. Minimally invasive tissue sampling (MITS) is an alternative to autopsy for identification of the cause of death (CoD). A formative research linked to pilot MITS implementation was conducted to document the perceptions and attitudes of the healthcare professionals and the barriers for implementation.

**Methods:**

This exploratory qualitative study conducted at a tertiary care hospital in New Delhi, India included the hospital staffs. In-depth interviews were conducted with the doctors, nurses and support staffs from pediatrics, neonatology, obstetrics and forensic medicine departments. Inductive data analysis was done to identify the emerging themes and codes.

**Results:**

A total of 26 interviews (doctors, *n* = 10; nurses, *n* = 9 and support staffs, *n* = 7) were conducted. Almost all professional and support staffs were positive about the MITS and its advantage for CoD identification including co-existing and underlying illnesses. Some opined conduct of MITS for the cases without clear diagnosis. All participants perceived that MITS would be acceptable for parents due to the non-disfigurement and preferred by those who had unexplained child deaths or stillbirths in past. The key factors for MITS acceptance were appropriate communication, trust building, involvement of senior doctors, and engagement of the counselor prior to deaths and training of the personnel. For implementation and sustenance of MITS, involvement of the institute authority and government stakeholders would be essential.

**Conclusions:**

MITS was acceptable for the doctors, nurses and support staffs and critical for better identification of the causes of death and stillbirths. The key facilitating factors and challenges for implementing MITS at the hospital in Indian context were identified. It emphasized on appropriate skill building, counseling, system organization and buy-in from institution and health authorities for sustenance of MITS.

## Plain English summary

Despite significant progress in child and maternal health indicators, India contributes the largest share of childhood deaths and stillbirths globally. For designing and implementing effective clinical and public health programs, adequate knowledge about the causes childhood deaths and stillbirths are essential. The currently available causes of death (CoD) and stillbirth are primarily derived from verbal autopsy (VA), a history and questionnaire based enquiry which has several limitations. Complete diagnostic autopsy is the best possible method for CoD identification, but with limited acceptability. Minimally invasive tissue sampling (MITS) technique has recently emerged as an alternative to autopsy. Before implementation, it was necessary to understand the perceptions, practices, acceptance and barriers among the healthcare professionals regarding MITS. An exploratory qualitative study was undertaken at a tertiary care hospital in New Delhi, India using in-depth interviews with different cadres of healthcare professionals from paediatrics, neonatology, obstetrics and forensic medicine departments. MITS was acceptable for almost all healthcare professionals and support staffs. They perceived MITS to be useful for identification of underlying causes of death and stillbirth, especially in cases of unexplained deaths or stillbirths. They perceived appropriate communication, trust building and involvement of senior doctors as the key factors for MITS acceptance by parents.

## Background

Globally, about 5.3 million under-five children and 2.6 million neonates died globally in 2018 [1]. About 2.6 million stillbirths also occur every year globally [2–4]. India was the largest contributor of under-five and neonatal deaths and stillbirths in 2018 [1]. About 20% of the global stillbirths occured in India [5]. Despite the significant progress in child and maternal health indicators for India, the decline in neonatal death and stillbirth rates are slower. Effective implementation of cost-effective interventions at scale with quality can prevent majority these childhood deaths [6]. As per National Health Policy (2017), India targets reducing the under-five and neonatal mortality rates to 23 and < 10 per 1000 livebirths by 2025 [7]. For designing and implementing effective interventions, adequate knowledge about the infectious and non-infectious causes of death (CoD) and stillbirths are critical.

The current information on the CoD in children is primarily based on verbal autopsy (VA), as many children die at community level or before reaching hospitals or any healthcare provider. Out of those who reach health facilities, the documentation of CoD is not adequate due to inadequate investigation and incomplete information about CoD in death declaration, often missing the coexisting illnesses or factors [8–12]. Complete diagnostic autopsy, although can provide critical information on CoD, its conduct is dismal for non-legal cases due to the sociocultural and religious norms and technical and infrastructural challenges [13–17]. The CoDs identified through VA have several limitations due to the non-specific clinical features in newborns and infants, inability to capture the co-existing and underlying problems [18–21]. VA is not much useful in identification of the cause(s) of stillbirths. The minimally invasive tissue sampling (MITS) technique involves detailed physical examination, imaging, targeted tissue sampling and processing for histopathologic, microbiological and other desired investigations. MITS has been observed to be efficient for CoD identification [22–24]. MITS is less invasive, non-disfiguring and time and cost efficient, but has results comparable to the standard autopsy for CoD [25–27]. MITS is being used in project mode in few countries. The formative researchers and early implementation experiences have revealed positive theoretical acceptability for MITS among parents, community members and religious leaders [28–32]. Acceptance for MITS among the healthcare providers at facility and community levels have been encouraging [33–36]. The acceptance of MITS among the families and community may vary across the countries due to the socio-cultural and religious contexts. The acceptance among the healthcare providers may also vary based on the experience, infrastructure, and sociocultural norms.

A pilot project was undertaken in India as part of the Child Health and Mortality Prevention Surveillance Network, to document the feasibility, acceptability, capacity building and standardization to determine the CoD. This pilot project included conduct of MITS to identify the CODs for under-five deaths including neonatal deaths and stillbirths. The healthcare professionals and hospital staffs interact closely with the parents and family members during the treatment and death declaration and are likely to influence the MITS acceptability. Thus, for conduct of MITS, it was important to understand the perception and attitudes of these key stakeholders. A formative research was conducted to understand the perceptions, practices, contextual factors and barriers for acceptance and conduct of MITS. The study protocol has been published earlier [37].

## Methods

### Study design and setting

This exploratory qualitative research adopted grounded theory technique. This study was conducted at Safdarjung Hospital (SJH), New Delhi, India, during December 2018 to January 2019.

### Study participants

The healthcare providers from paediatrics (neonatology and children wards), obstetrics and the support staffs from SJH were purposively sampled. The healthcare providers included doctors (junior residents, senior residents and faculty) and nurses from different wards/units. The faculty and mortuary staff from forensic medicine department were also included.

### Data collection

Guides for IDIs were prepared covering the domains and issues to be explored based on the literature review (Supplementary document). The IDI guides had open-ended questions that explored the perceptions regarding the cause(s) of death in children or stillbirth, attitudes and opinions about the conduct, benefit, acceptance and challenges for MITS. During the interview, before asking about MITS, the interviewer described the MITS procedure components. A team of investigator and trained research staffs conducted the interviews at places convenient for the participants. During the interview, free flow of the information was encouraged with use of probes and additional issues were discussed, as they emerged. The IDIs were conducted in either local language (Hindi) or English as convenient for the participant and audio-recorded with consent. The research team took detailed field notes and noted the verbal and non-verbal languages and expressions. The interviews usually lasted for about 45–60 min.

### Data handling and analysis

The audio records and field notes were transcribed verbatim and then translated into English, if needed. An independent member checked for the completeness and correctness of the transcriptions and quality of translation with the audio-records and field notes. The data was entered using INCLEN Qualitative Data Analysis Software (IQDAS), which allows data entry, organization and retrieval for analysis in Indian languages and English. The entered data were checked completeness and correctness. The data were saved into the server and daily back up was taken. The inductive data analysis followed the steps: free listing, domain identification, coding, and cross tabulation. The emerged codes and themes were discussed periodically and the discrepancies were resolved. From the codes, the axial codes were identified followed by grouping into selective codes and organized under key themes. An iterative process was adopted for the transcript reading, coding and theme identification till consensus was reached. The themes across the participant categories were triangulated for consistencies and diversions. The findings were summarized using semi-quantitative qualifiers: very few (< 10%), some (10–24%), about half (25–49%), majority (50–75%), most (76–89%) and almost all (> 90%).

### Ethical considerations

Ethical approval for the study was obtained from the participating institute ethics committees (INCLEN Ethics Committees, Ref: IIEC 51 and Institutional Ethics Committee, VMMC and Safdarjung Hospital, Ref: IEC/SJH/VMMC/Project/August-2017/1000). The study participants were recruited after obtaining written informed consent including permission for audio recording and use of anonymized quotes. The interviews were conducted in isolation and no one other than the participant and interviewer was present during the process. Confidentiality and anonymity of participants were assured.

## Results

A total of 26 IDIs were conducted with doctor (*n* = 10), nurses (*n* = 9) and support staffs (*n* = 7) (Table 1). All the participants approached consented and participated. Based on the data analysis, the key thematic areas identified were: (1) perceived benefit; (2) acceptance by families; (3) hospital and system readiness and (4) factors influencing implementation. The findings are presented below.

**Table 1:** The stakeholders participated in the study

Sl	Stakeholder category	Number
1	Profession	
*1.1*	Doctors, n	10
	- Pediatrics (Junior resident, Senior resident and Faculty, 1 each)	3
	- Neonatology (Junior resident, Senior resident and Faculty, 1 each)	3
	- Obstetrics (Junior resident, Senior resident and Faculty, 1 each)	3
	- Forensic medicine (Faculty)	1
*1.2*	Nurses, n	9
	- Pediatrics (from different wards)	3
	- Neonatology	3
	- Obstetrics (labour rooms and postnatal ward)	3
*1.3*	Support staffs, n	7
	- Pediatrics (from different wards)	2
	- Neonatology	2
	- Obstetrics (labour rooms and postnatal ward)	2
	- Mortuary	1
*2*	Gender, n	26
	- Female	19
	- Male	7
*3*	Age group, median (range) in years	
	- Faculty members	47 (45–52)
	- Resident doctors	28 (26–29)
	- Nurses	45 (37–52)
	- Support staffs	42 (34–48)
*4*	Service tenure, median (range) in years	
	- Faculty members	15 (13–20)
	- Residents	3 (2–5)
	- Nurses	16 (13–22)
	- Support staffs	15 (12–18)

### Perceived benefit

#### Better and accurate diagnostic ability

Most healthcare professionals and support staffs favoured MITS considering that it might offer better and accurate diagnosis. The paediatricians expressed that in several cases they were unable to make conclusive diagnosis or CoD due to shorter time of hospitalisation or critical condition and limited opportunity for the investigations. The obstetricians mentioned that causes of stillbirths remain unknown in most scenarios. The detailed examination, imaging, body fluids and tissue samples collected by needle biopsies would potentially allow better diagnosis. The microbiological, pathological and molecular diagnostic tests could provide valuable information for determining the causes of death and stillbirth. The forensic expert indicated that the acceptance for autopsy was very low and in many medicolegal cases arguments happen with the families over the body disfigurement and time taken.

#### Identification of less common and co-existing illnesses

Doctors perceived that MITS has potential to allow identification of less common, coexisting and underlying illnesses. The paediatricians opined that it would be very useful in neonatal deaths, where sepsis is assigned as CoD in most cases. All informants felt that MITS would be useful to improve the knowledge and input for the treatment and programs.

#### General or selective approach

The professionals had different opinions on case selection for MITS. While majority preferred conduct of MITS in all cases where the diagnosis or CoD remains unknown, some felt that the cases be selected by the treating doctors, as per the need. Few doctors perceived MITS to be futile in cases with clear diagnosis or with major underlying disease or malformation.

### Potential acceptance by parents

#### Acceptance of MITS by parents

The health professionals and staffs indicated that MITS in view of the no disfigurement and organ retrieval, MITS could be acceptable for the families. Most of them perceived that the willingness for MITS may depend on the perceived benefit by the families. They felt that the families with past child death or bad obstetric history might be more willing for MITS than others. Majority of the informants also felt that the education, sociocultural and religious factors would influence the acceptance for MITS. Few doctors felt that the MITS findings might be counterproductive and trigger aggression in some cases, where the family don’t accept the death or agree with the CoD.

#### When to approach for consent

Almost all opined that the family may be approached for consent after few minutes of death declaration, as some time may be needed by them to accept the death and come to a state of potential understanding. Regarding the person to be approached for consent, almost all were unanimous that the father, grandfather or other male member present in the hospital should be approached. According to the obstetricians and nurses, the stillbirths were usually declared to the husband or family members and not the woman directly. The nurse and support staffs felt that the parents and family who lost their child or pregnancy would be in a grief state and it would be challenging to approach them at that time.

*“Most of the time, they will not give consent, because you can imagine that most of the doctor’s face violence and verbal abuse, imagine if you will say that we are going to take samples out of body then, maybe that will counter benefit than beneficial. Then they will ask that what is benefit of that, they are not getting any benefit, maybe hospital is getting any benefit, and their baby has died.” (Doctor-Pediatrics)*

*“It (MITS) will help, but I don’t think so people will give permission to that. It will depend on, I will tell you, who accept death and who don’t.” (Doctor-Pediatrics)*

### Hospital and system readiness

#### Readiness of healthcare professionals

Almost all of the participants mentioned that the hospital was already overloaded and the professionals were overstretched with the existing workload. According to them the MITS would be a time taking procedure considering the counselling, consent and conduct of the procedure. According to them, separate teams would be needed for obtaining the consent and conduct of MITS. Additionally the other departments and teams like microbiology, pathology and radiology should be involved from the preparatory phase. They perceived that if it has to be conducted in a project mode, it might be feasible, but for routine services, additional planning would be needed.

*“The workload will increase, if the present doctors have to perform these tests. It would be better if other team is allotted to perform MITS procedure.” (Doctor-Pediatrics)*

*“The workload on doctors will increase. Here staff nurses don’t do any sampling. Occasionally put the IV cannula. All invasive procedures are done by doctors. By this the pressure on doctors will increase.” (Nurse-Pediatrics)*

#### Skills and training

The health professionals opined that appropriate training of the doctors, nurses and counsellors shall be needed for obtaining consent and conduct of MITS. They felt that the skills of the counselling team would be critical to interact with families for obtaining consent. The doctors opined that appropriate orientation and protocol needs to be prepared for all the involved procedures and departments for appropriate function and outcome. Almost all of the participants expressed that the declaration of stillbirth and death affected them and their emotions, although they have learnt over time how to handle it. They opined that there should be training sessions for young doctors and nurses on how to deal with the deaths and related counselling.

*“We don’t get any specific teaching and reading materials on the part of counseling. It is self-reading, observational, sometimes told in classrooms.” (Doctor-Pediatrics)*

*“It is very bad for us, no matter how rigid we appear on the face, but in the night when we go home, there is a striking force that child expired on our duty. Many times I am not able to sleep because of this fact.” (Doctor-Pediatrics)*

#### Infrastructure, equipment and cost implications

The doctors stressed on appropriate infrastructure creation, equipment and supplies for conduct of MITS, which should be funded by government or from project, as appropriate. The participants opined that the MITS procedure should be free of cost for the family, as additional cost would be a challenge for obtaining consent. The nurses and support staffs felt that a separate area for MITS be prepared, which could be accessed easily and allow waiting of parents and family members. Most of the doctors felt that if the institute leadership and government decision makers are convinced, cost would not be a concern. According to them, if huge investments are needed, then it might pose challenge for sustaining beyond the project period.

### Factors influencing implementation

#### Effective communication and trust

The participants were unanimous about the need for transparent and frequent communication with the parents to build trust and confidence. According to most of the participants, the counsellors should preferably engage with the families before the death to provide some time for rapport and confidence building. The quality and ability of the counsellor was indicated as critical by majority of the doctors and nurses. They also felt that senior doctor’s presence could improve acceptance and consent by families. Some of the nurses and support staffs felt that after conduct of the MITS, the family should be shown the body before handover. Few participants opined that training would be needed for all the staffs on appropriate communication with the patients and families for effective implementation.

*“No, I think it is important. I have not taken any training in this field, so I sometimes explain them. We don’t know the proper way. Whatever way I explain they listen to it, they don’t react abnormally, but still I think it can be improved.” (Doctor–Obstetrics)*

#### Conduct of the MITS by trained person

The doctors felt that in view of the importance and value, a trained person should conduct MITS and should not be taken for granted. Some of the professionals were apprehensive that MITS, if conducted by the junior residents might be useless.

*“Availability of trained person and space is challenge. We don’t have any trained person, we don’t know who is going to do it and where they are going to do it. You do it and then put the things in reverse gear.” (Doctor –Obstetrics)*

#### Timing for counselling and conduct of MITS

Almost all of the participants highlighted that MITS should be conducted as quickly as possible to minimise the denials. Most of them were unsure about the appropriate timing to approach for consent and felt that the team on ground may decide the timing. Almost all of the participants opined that the families would like to take the body and leave as soon as possible after the death. Some felt that once the family members appear settled and in a state to discuss, they should be approached for consent. Some of the respondents felt that the deaths occurring towards the later part of the day or at night might be challenging, as the families would like to move quickly and or might not have the decision maker present. The health professionals mentioned that a quicker conduct of MITS, within 1–2 h would be acceptable for the families and delay in procedure would reduce acceptance.

#### Sharing the findings with parents and family

The doctors and nurses felt that the findings of MITS should be shared with the parents and family at the earliest possible. According to them, if delayed, it might not be considered useful for them. Also they felt that the treating senior doctor should disclose and counsel the parents.

#### Institute and government facilitation

The doctors were of opinion that for effective implementation, there is need for buy-in and facilitation from the institute leadership and government to ensure funding for the infrastructure, manpower and supporting cost. Additionally for keeping the relevant stakeholders and departments engaged in the implementation, the support and facilitation from higher officials shall be critical.

## Discussion

This is the first study from India to our best of knowledge documenting the perceptions and attitudes of the health professionals and support staffs on MITS and related issues. When autopsy is not acceptable to the families, the MITS is an alternative to know causes of death and stillbirth. But for conduct and sustenance the MITS within the existing system, the view of healthcare professionals and support staffs are important. We found that the hospital staffs were positive about acceptance of MITS among the parents, if offered to know the CoD in children and stillbirths. According to them, tThe factors in favor of MITS acceptance were no disfigurement and organ removal. They opined that the acceptance would be more among the parents with past experience of child death or bad obstetric history. These factors were similar to those identified in studies from United Kingdom, Pakistan, Gabon, Kenya, Mali, Mozambique and Kenya [13, 33–35, 38].

The study explored the factors that might influence implementation of MITS in a tertiary care hospital in India. Overall, the professionals and staffs expressed MITS to have value addition for identification of causes of death and stillbirth, which shall improve the treatment and health programs. Higher possibility of identification of underlying and uncommon causes with better diagnostic accuracy were considered as the benefits of MITS, which were similar to the other studies [34, 35, 39].

A formative research in Pakistan observed possible high theoretical MITS acceptance among the parents, especially by those who have experienced multiple neonatal deaths, stillbirths or miscarriages. The trust on healthcare providers and hospital, effective counseling and promptness in conducting MITS were identified as the influencing factors for the acceptance. The participants also opined that appropriate healthcare providers should be identified for training in MITS to retain interest [34]. These observations were similar to the findings from our study.

Healthcare providers from Mozambique had negative opinion about complete autopsy, as it was not easy to perform, procedures involved, unpleasantness, physical and mental discomfort for the performer and required specialized training and competency to conduct. Those who had experience of conducting autopsy found the minimally invasive autopsy (MIA) as simple, easy and quick technique that could be done anywhere and by anyone after appropriate training even in the absence of formal pathology facilities. They also found the MIA to be cleaner, less messy, without unpleasant smell and higher family compliance. But some healthcare providers felt that the yield and productivity would be inferior to the complete autopsy [35]. The findings from our study had similar observations.

A survey among healthcare providers in United Kingdom reported higher acceptability for MIA compared to autopsy across the ethnic and religious group. Some of the respondents had concerns about the accuracy of the MIA. Some of the healthcare providers had concern about the acceptance among Indian, Asian or Arab ethnicity in view of their religious and cultural practices [40].

However some professionals felt that MITS should be reserved for patients with no or incomplete diagnosis. These observations were similar to the observations from other studies [34, 35].

Several studies have identified the challenges for obtaining consent for postmortem examination and procedures for children [13, 32, 41, 42]. A review of 34 reports on acceptance of autopsy and MIA identified seven themes describing the barriers and six facilitating themes for postmortem acceptance among parents. The seven barrier themes were dislike of invasiveness, practicalities of the procedure, organ retention issues, protective parenting, communication and understanding, religion and culture and professional or organisational factors. The six facilitating themes for consent included desire for information, contribution to research, coping and well-being, respectful care, minimally invasive procedure, and policy and practice [13]. The participants mentioned the challenges related to acceptance of postmortem by the families including their willingness to know the CoD, disfigurement, delay in the process and final rituals and religious norms. The participants perceived the advantages and lesser concerns compared to complete autopsy would facilitate its acceptance by the families.

To encourage the acceptance of MITS, the participants indicated the importance of appropriate communication, effective counselling and explanation by the health professionals. The study found that the counselors and health professionals, especially the younger doctors and nurses, should be trained for effective communication and counselling. It was also found that the counselors should be involved prior to the death along with the treating team to build rapport. The study identified that counseling by the senior doctors and the staffs with good rapport with the parents would improve the chance of acceptance. These findings were similar to the other studies on MITS and postmortem autopsy [34, 41, 42]. Although no definite time window to approach for consent was identified, the participants felt that it should be decided by the counselor and doctor. It was found that the MITS should be conducted quickly to minimize logistic hassles for families. The participants suggested early feedback to the parents on findings for appropriate action and retain the confidence on hospital.

For implementation of MITS, several system level challenges were identified including manpower shortage, additional work for the existing staffs, infrastructural and equipment support, cooperation from the concerned departments for processing the samples and cost. It also found that for sustenance beyond the project period, engagement of the institute authority and government decision makers are needed.

The opinions from different categories of healthcare providers from the concerned departments was the strength. The study had some limitations. The results represent the views and attitudes of the healthcare professionals and staffs from one hospital in India, which may differ from the opinion of health professionals from other parts of the country. The MITS acceptability explored was hypothetical, prior to the implementation, which may change with real experience. Future research may be undertaken to document the changes in the views and attitudes of the healthcare professionals with implementation of MITS including the facilitators and barriers.

## Conclusion

Overall, MITS was viewed positively and acceptable to the healthcare professionals and staffs at the tertiary care hospital. The healthcare providers perceived MITS to be acceptable to the parents and assist in better diagnosis and appropriate treatment. The study identified several concerns and challenges for the professionals, health system and administration for effective implementation and sustenance in Indian context. The study underscored the importance of appropriate communication, trust building and counselling for MITS acceptance by the parents. Appropriate manpower engagement, training, infrastructural support and administrative facilitation would help successful implementation and sustenance of MITS.

## Supplementary information

**Additional file 1.**

## Data Availability

The datasets used and analyzed during the current study are available from the corresponding author on reasonable request.

## Abbreviations

CoD: Causes of death
IDI: In-depth interview
IQDAS: INCLEN Qualitative Data Analysis Software
MITS: Minimally invasive tissue sampling
NICU: Newborn intensive care unit
SJH: Safdarjung Hospital
SNCU: Sick newborn care unit
VA: Verbal autopsy
