# Supplementary material for: Perceptions of the healthcare providers regarding acceptability and conduct of minimal invasive tissue sampling (MITS) to identify the cause of death in under-five deaths and stillbirths in North India: a qualitative study
Source: BMC Health Serv Res. 2020 Sep 4;20:833. doi: 10.1186/s12913-020-05693-6 (PMC7472696; doi:10.1186/s12913-020-05693-6)
Supplement: Supplementary file 1 — Additional file 1. [file 12913_2020_5693_MOESM1_ESM.docx]

**Key Informant In-depth interview guide**

**Health care provider- Pediatrician**

1. **Basic demography**
   1. Designation
   2. Total years of service
   3. Total years in current position

**Service delivery and communication**

1. You come across several patients on daily basis. How do you inform and counsel the parents/ family members of children/newborn under your care?

(Probe: Approach immediately after hospitalization, during the course of illness and discharge)

1. You come across several sick patients, who are at high risk for death. How do you inform and counsel the parents/ family members of critically ill children/newborn under your care?

(Probe: How does the approach for critically ill children differ from the regular non-critically ill children, time devoted, frequency, person counseling/discussing with family)

1. What has been the hardest part about being a doctor treating sick children/newborn?

(Probe: informing/counseling the parents and family members, handling the deaths, etc)

**Death and related procedures**

1. Several of children/newborn under your care die. Please describe the death declaration process usually followed at the hospital.

(Probe: How is the death declaration done, place of declaration, time taken, whom primarily targeted, usual reaction from families)

1. Please let us know about your personal experience of last death declaration in patient under your care?

(Probe: Mode of declaration, time taken, family members present, reaction from family members)

1. What are the challenges encountered by you regarding death declaration in patients under your care?

(Probe: explaining and convincing parents/family members, handling reactions, time needed, workload and other competing priorities, support from other colleagues/senior and staffs, security)

1. How your juniors (including trainees), co-workers, and other hospital staffs usually support around death of a child under your care?

(Probe: Who supports, what type of support given, any expectations)

1. How do you handle the reaction/response of parents/family members related to death?

(Probe: explanation, seeking support from senior/colleagues, security)

1. What is your view about the causes of death, declared and exact/underlying cause/etiology for the children dying in the hospital or brought dead to the hospital?

(Probe: approach to declaration, past records, mention of cause of death, effort for autopsy)

1. What has been your experience with autopsy, post-mortem biopsy after death?

(Probe: Are these being done, benefit, action based on the findings)

*You might have heard about Minimally Invasive Tissue Sampling (MITS). MITS involves collection of tissue samples similar to biopsy and body fluids from a deceased patient for investigations. The samples and fluids collection are subjected for various tests to identify the cause(s) of death.*

1. What is your view about MITS being used as a mode of identifying cause(s) of death in children/ newborn in your hospital?

(Probe: feasibility, acceptability, perceived benefit, workload)

1. In your view, how the parents/ family members can be approached and convinced for MITS?

(Probe: which health staff should approach, which family member should be approached, timing for informing, mode of communication, place of informing)

1. In your view, what could be the expectations of parents/family members if they agree for MITS?

(Probe: detailed report, convey of the findings, time, cost/money)

1. What are the potential challenges/barriers for autopsy & MITS in the hospital setting?

(Probe: procedural, space, manpower, workload, time needed, cost)

1. Any other comment/ suggestions.

Thank the respondent for his/her critical contribution.

**Key Informant In-depth interview guide**

**Health care provider- Obstetrician**

1. **Basic demography**
   1. Designation
   2. Total years of service
   3. Total years in current position

**Service delivery and communication**

1. You come across several pregnant women delivering at your hospital on daily basis. How do you inform and counsel the pregnant woman and her husband and family members under your care?

(Probe: Approach immediately after hospitalization, during hospitalization and discharge)

*You come across several pregnant women regularly. While many of the deliveries end with live births, some of the pregnancies end with stillbirths. Few pregnancies also end with intrauterine deaths. Majority of the stillbirths and intrauterine deaths are not predictable in advance.*

1. How do you inform and counsel the pregnant woman and husband or family members about the risk of stillbirth and intrauterine death?

(Probe: predictability, approach for high-risk pregnancies, time devoted, frequency, mode of counseling/discussion with family)

1. What has been the hardest part about being an obstetrician serving pregnant women?

(Probe: informing/counseling the woman, husband and family members, handling the stillbirth, intrauterine deaths)

**Stillbirth or Intrauterine death and related procedures**

*Some of the pregnancies end with stillbirth or intrauterine death in this hospital under your care.*

1. How do you declare the outcome in such situations?

(Probe: How is the declaration done, place of declaration, time taken, whom primarily targeted, usual reaction from families)

1. Please let us know about your personal experience of last stillbirth declaration in a patient under your care?

(Probe: Mode of declaration, time taken, family members present, reaction from family members)

1. What are the challenges encountered by you regarding stillbirth declaration in pregnant women under your care?

(Probe: explaining and convincing the women/husband/family members, handling reactions, time needed, workload and other competing priorities, support from other colleagues/senior and staffs, security)

1. How your juniors (including trainees), co-workers, and other hospital staffs usually support around stillbirth of a pregnant woman under your care?

(Probe: Who supports, what type of support given, any expectations)

1. How do you handle the reaction/response of woman, husband and family members related to stillbirth?

(Probe: explanation, seeking support from senior/colleagues, security)

1. What is your view about the causes of stillbirth and intrauterine death, declared and exact/underlying cause/etiology?

(Probe: approach to declaration, past records, mention of cause of death, effort for autopsy)

1. What has been your experience with perinatal autopsy for stillbirth and/or intrauterine death?

(Probe: Are these being done, benefit, action based on the findings)

*You might have heard about Minimally Invasive Tissue Sampling (MITS). MITS involves collection of tissue samples similar to biopsy and body fluids from a deceased patient for investigations. The samples and fluids collection are subjected for various tests to identify the cause(s) of death.*

1. What is your view about MITS being used as a mode of identifying cause(s) of death in stillbirth and intrauterine deaths in your hospital?

(Probe: feasibility, acceptability, perceived benefit, workload)

1. In your view, how the parents/family members can be approached and convinced for MITS?

(Probe: which health staff should approach, which family member should be approached, timing for informing, mode of communication, place of informing)

1. In your view, what could be the expectations of parents/family members if they agree for MITS?

(Probe: detailed report, convey of the findings, time, cost/money)

1. What are the potential challenges/barriers for autopsy & MITS in the hospital setting?

(Probe: procedural, space, manpower, workload, time needed, cost)

1. Any other comment/ suggestions.

Thank the respondent for his/her critical contribution.

**Key Informant In-depth interview guide**

**Health care provider- Nurse in Pediatrics/Neonatology unit**

1. **Basic demography**
   1. Designation
   2. Total years of service
   3. Total years in current position

**Service delivery and communication**

1. You come across several patients on daily basis. How are the parents/ family members of children/newborn in this ward/unit informed/counseled about the illness and condition?

(Probe: immediately after hospitalization, during the course of illness and discharge)

1. You come across several sick patients, who are at high risk for death. How are the parents/ family members of critically ill children/newborn in this ward/unit informed/counseled about the illness and condition?

(Probe: How does the approach for critically ill children differ from the regular non-critically ill children, time devoted, frequency, person counseling/discussing with family)

1. What has been the hardest part about being a nurse caring sick children/newborn?

(Probe: informing/counseling the parents and family members, handling the deaths, etc)

**Death and related procedures**

1. Several of children/newborn whom you care die. Please describe the death declaration process usually followed in this ward/unit.

(Probe: How is the death declaration done, place of declaration, time taken, whom primarily targeted, usual reaction from families)

1. Please let us know about your personal experience of last death declaration in patient you cared?

(Probe: Mode of declaration, time taken, family members present, reaction from family members)

1. What are the challenges encountered by you and the doctors regarding death declaration in patients you care?

(Probe: explaining and convincing parents/family members, handling reactions, time needed, workload and other competing priorities, support from doctors, other colleagues/senior and staffs, security)

1. How the senior doctors/consultants, residents, other nurses, and other hospital staffs usually support around death of a child whom you care?

(Probe: Who supports, what type of support given, any expectations)

1. How do you handle the reaction/response of parents/family members related to death?

(Probe: explanation, seeking support from senior/colleagues, security)

1. What is your view about the causes of death, declared and exact/underlying cause/etiology for the children dying?

(Probe: approach to declaration, past records, mention of cause of death, effort for autopsy)

*You might have heard about Minimally Invasive Tissue Sampling (MITS). MITS involves collection of tissue samples similar to biopsy and body fluids from a deceased patient for investigations. The samples and fluids collection are subjected for various tests to identify the cause(s) of death.*

1. What is your view about MITS being used as a mode of identifying cause(s) of death in children/ newborn?

(Probe: feasibility, acceptability, perceived benefit, workload)

1. In your view, how the parents/ family members can be approached and convinced for MITS?

(Probe: which health staff should approach, which family member should be approached, timing for informing, mode of communication, place of informing)

1. In your view, what could be the expectations of parents/family members if they agree for MITS?

(Probe: detailed report, convey of the findings, time, cost/money)

1. What are the potential challenges/barriers for autopsy & MITS in the hospital setting?

(Probe: procedural, space, manpower, workload, time needed, cost)

1. Any other comment/ suggestions.

Thank the respondent for his/her critical contribution.

**Key Informant In-depth interview guide**

**Health care provider- Nurse in the delivery room or postnatal ward (Obstetrics)**

1. **Basic demography**
   1. Designation
   2. Total years of service
   3. Total years in current position

**Service delivery and communication**

1. You come across several pregnant women delivering at your hospital on daily basis. How are the pregnant woman, her husband and family members in this ward/unit informed/counseled about the status, problem and outcome?

(Probe: immediately after hospitalization, during the course of illness and discharge)

*You come across several pregnant women regularly. While many of the deliveries end with live births, some of the pregnancies end with stillbirths. Few pregnancies also end with intrauterine deaths. Majority of the stillbirths and intrauterine deaths are not predictable in advance.*

1. How the pregnant woman, her husband or family members are informed/counseled about the risk of stillbirth and intrauterine death?

(Probe: predictability, approach for high-risk pregnancies, time devoted, frequency, mode of counseling/discussion with family)

1. What has been the hardest part about being a nurse caring/serving obstetrician serving pregnant women?

(Probe: informing/counseling the woman, husband and family members, handling the stillbirth, intrauterine deaths)

**Stillbirth or Intrauterine death and related procedures**

*Some of the pregnancies end with stillbirth or intrauterine death in this hospital.*

1. How are the stillbirths and intrauterine deaths declared in your unit/hospital?

(Probe: How is the declaration done, place of declaration, time taken, whom primarily targeted, usual reaction from families)

1. Please let us know about your personal experience of last stillbirth declaration in a patient in this unit?

(Probe: Mode of declaration, time taken, family members present, reaction from family members)

1. What are the challenges encountered regarding stillbirth declaration in pregnant women in this unit/ hospital?

(Probe: explaining and convincing the women/husband/family members, handling reactions, time needed, workload and other competing priorities, support from other colleagues/senior and staffs, security)

1. How the senior doctors/consultants, residents, other nurses, and other hospital staffs usually support around stillbirth of a pregnant woman in your unit?

(Probe: Who supports, what type of support given, any expectations)

1. How are the reactions/responses of woman, husband and family members related to stillbirth handled?

(Probe: explanation, seeking support from senior/colleagues, security)

1. What is your views about the causes of stillbirth and intrauterine death, declared and exact/underlying cause/etiology?

(Probe: approach to declaration, past records, mention of cause of death, effort for autopsy)

*You might have heard about Minimally Invasive Tissue Sampling (MITS). MITS involves collection of tissue samples similar to biopsy and body fluids from a deceased patient for investigations. The samples and fluids collection are subjected for various tests to identify the cause(s) of death.*

1. What is your view about MITS being used as a mode of identifying cause(s) of death in stillbirth and intrauterine deaths in your hospital?

(Probe: feasibility, acceptability, perceived benefit, workload)

1. In your view, how the parents/family members can be approached and convinced for MITS?

(Probe: which health staff should approach, which family member should be approached, timing for informing, mode of communication, place of informing)

1. In your view, what could be the expectations of parents/family members if they agree for MITS?

(Probe: detailed report, convey of the findings, time, cost/money)

1. What are the potential challenges/barriers for autopsy & MITS in the hospital setting?

(Probe: procedural, space, manpower, workload, time needed, cost)

1. Any other comment/ suggestions.

Thank the respondent for his/her critical contribution.

**Key Informant In-depth interview guide**

**Health care provider- Support staff- Pediatrics/Neonatology unit**

1. **Basic demography**
   1. Designation
   2. Total years of service
   3. Total years in current position

**Service delivery and communication**

1. You come across several patients on daily basis. How are the parents/ family members of children/newborn in this ward/unit informed/counseled about the illness and condition?

(Probe: immediately after hospitalization, during the course of illness and discharge)

1. You come across several sick patients, who are at high risk for death. How are the parents/ family members of critically ill children/newborn in this ward/unit informed/counseled about the illness and condition?

(Probe: How does the approach for critically ill children differ from the regular non-critically ill children, time devoted, frequency, person counseling/discussing with family)

1. What has been the hardest part about being a staff caring sick children/newborn?

(Probe: handling death, day-to-day care)

**Death and related procedures**

1. Several of children/newborn in this ward/unit die. You might have observed death declaration for several children/newborn. Please describe the death declaration process usually followed in this ward/unit

(Probe: How is the death declaration done, place of declaration, time taken, whom primarily targeted, usual reaction from families)

1. Please let us know about your personal experience of last death declaration you observed?

(Probe: Mode of declaration, time taken, family members present, reaction from family members)

1. In your view, what are the challenges encountered by the doctors/nurses and other staffs while death declaration in patients?

(Probe: explaining and convincing parents/family members, handling reactions, time needed, workload, security)

1. How the senior doctors/consultants, residents, other nurses, and other hospital staffs usually support each other around death of a child/newborn?

(Probe: Who supports, what type of support given, any expectations)

1. In your view, how are the parents/family members of the child who is dying/dead prepared for death declaration?

(Probe: informing parents/family in advance, explanation)

1. How are the reaction/response of parents/family members related to death handled?

(Probe: crying, outrage/fight, question)

1. What is your view and experience about the autopsy for children/newborn dying in this hospital?

(Probe: Are these being done, benefit, action based on the findings)

*In some instances, autopsy/ post-mortem is not possible due to various reasons. To find out the exact cause of death and underlying disease, some tissue biopsy (collection of very small sample using needles) and fluids (blood, urine, etc.) from the body, as done for biopsy. This procedure is called as Minimally Invasive Tissue Sampling (MITS).*

1. What is your view about MITS being used as a mode of identifying cause(s) of death in children/ newborn?

(Probe: feasibility, acceptability, perceived benefit, workload)

1. In your view, how the parents/ family members can be approached and convinced for MITS?

(Probe: Who/which health staff should approach, which family member should be approached, timing for informing, mode of communication, place of informing)

1. In your view, what could be the expectations of parents/family members if they agree for MITS?

(Probe: detailed report, convey of the findings, time, cost/money)

1. What are the potential challenges/barriers for autopsy & MITS in the hospital setting?

(Probe: procedural, space, manpower, workload, time needed, cost)

1. Any other comment/ suggestions.

Thank the respondent for his/her critical contribution.

**Key Informant In-depth interview guide**

**Health care provider- Support staff at the Hospital (Pediatrics unit and Delivery room)**

1. **Basic demography**
   1. Designation
   2. Total years of service
   3. Total years in current position

**Service delivery and communication**

1. You come across several pregnant women delivering at your hospital on daily basis. How are the pregnant woman, her husband and family members in this ward/unit informed/counseled about the status, problem and outcome?

(Probe: immediately after hospitalization, during the course of illness and discharge)

*You come across several pregnant women regularly. While many of the deliveries end with live births, some of the pregnancies end with stillbirths. Few pregnancies also end with intrauterine deaths. Majority of the stillbirths and intrauterine deaths are not predictable in advance.*

1. How the pregnant woman, her husband or family members are informed/counseled about the risk of stillbirth and intrauterine death?

(Probe: predictability, approach for high-risk pregnancies, time devoted, frequency, mode of counseling/discussion with family)

1. What has been the hardest part about being a staff caring/serving pregnant women?

(Probe: informing/counseling the woman, husband and family members, handling the stillbirth, intrauterine deaths)

**Stillbirth or Intrauterine death and related procedures**

*Some of the pregnancies end with stillbirth or intrauterine death in this hospital. You might have observed death declaration for several children/newborn.*

1. How are the stillbirths and intrauterine deaths declared in this unit/hospital?

(Probe: How is the declaration done, place of declaration, time taken, whom primarily targeted, usual reaction from families)

1. Please let us know about your personal experience of last stillbirth declaration in a patient in this unit?

(Probe: Mode of declaration, time taken, family members present, reaction from family members)

1. In your view, what are the challenges encountered by the doctors/nurses and other staffs while declaring the stillbirths?

(Probe: explaining and convincing the women/husband/family members, handling reactions, time needed, workload, support from other staffs, security)

1. How the senior doctors/consultants, residents, other nurses, and other hospital staffs usually support each other around a stillbirth?

(Probe: Who supports, what type of support given, any expectations)

1. In your view, how are the parents/family members of the stillbirth dead prepared for the declaration?

(Probe: informing the woman, husband/family, mode of declaration)

1. How are the reactions/responses of woman, husband and family members related to stillbirth handled?

(Probe: explanation, seeking support from senior/colleagues, security)

1. What has been your experience with perinatal autopsy for stillbirth and/or intrauterine death?

(Probe: Are these being done, benefit, action based on the findings)

*In some instances, autopsy/ post-mortem is not possible due to various reasons. To find out the exact cause of death and underlying disease, some tissue biopsy (collection of very small sample using needles) and fluids (blood, urine, etc.) from the body, as done for biopsy. This procedure is called as Minimally Invasive Tissue Sampling (MITS).*

1. What is your view about MITS being used as a mode of identifying cause(s) of the stillbirths and intrauterine deaths in this hospital?

(Probe: feasibility, acceptability, perceived benefit, workload)

1. In your view, how the parents/family members can be approached and convinced for MITS?

(Probe: which health staff should approach, which family member should be approached, timing for informing, mode of communication, place of informing)

1. In your view, what could be the expectations of parents/family members if they agree for MITS?

(Probe: detailed report, convey of the findings, time, cost/money)

1. What are the potential challenges/barriers for autopsy & MITS in the hospital setting?

(Probe: procedural, space, manpower, workload, time needed, cost)

1. Any other comment/ suggestions.

Thank the respondent for his/her critical contribution.
